# Diagnosis and Treatment of Multiple Intestinal Angioectasias: A Case Report

**DOI:** 10.4021/gr298w

**Published:** 2011-03-20

**Authors:** Lidia Santarpia, Maria Carmen Pagano, Rosario Cuomo, Franco Contaldo, Fabrizio Pasanisi

**Affiliations:** a Internal Medicine and Clinical Nutrition, Department of Clinical and Experimental Medicine, Federico II University, Naples, Italy; b Gastroenterology and Hepatology, Department of Clinical and Experimental Medicine, Federico II University, Naples, Italy

**Keywords:** Intestinal angioectasias, Intestinal bleeding, Octreotide

## Abstract

The diagnostic course and management of a severe anemia due to recurrent bleeding from colonic angioectasias have been described. A 63-year-old man with chronic heart and renal failure, hypertension and diabetes presented severe anemia requiring transfusion. Anemia recurred and did not recover despite intravenous iron, folate and B_12_ vitamin supplementation, associated with eritropoietin administration. A bleeding angiodysplasia was finally diagnosed and long-acting octreotide prescribed, obtaining increased hemoglobin levels in the time.

## Introduction

Obscure gastrointestinal bleeding is a chronic occult or overt bleeding from the intestinal tract that remains unexplained by standard upper and lower endoscopic and radiological gastrointestinal investigations [1]. Causes are age dependent, with angioectatic vessels and nonsteroidal anti-inflammatory drug use being most common in patients older than 40 years [2].

Intestinal angioectasias (AE) can cause acute, recurrent bleeding or chronic anemia resulting in very frequent hospitalizations and transfusions [3]. Those lesions are often multiple, localized in sites inaccessible to endoscopy and have a high risk of recurrence despite an appropriate surgical or endoscopic treatment [4].

The current standard of endoscopic treatment is not always successful, even when lesions are accessible to gastrocolonoscopy, and surgery may be too invasive, in particular in the presence of comorbidities. In this context, adjunctive or even primary medical therapy such as octreotide may have a role [5].

## Case Report

A 63-year-old man working as a nurse in our Department, during his Sunday working day-shift, performed a routine hemato-biochemical exam due to a persisting sensation of weakness. The results revealed a severe anemia (Hb = 5.4 g/dL). He was promptly hospitalized and transfused with 2 unities of concentrated red blood cells. At the anamnesis he referred sporadic episodes of dark feces emissions since 15 - 20 days and anti-inflammatory drugs use due to a knee pain. The patient’s history was significant for obesity (weight 115 kg, BMI 42.27 kg/m^2^), type 2 diabetes, hypertension, and chronic heart and renal insufficiency. He was on therapy with metformin 1000 mg x 3/day, ramipril + hydrocholorotiazide 5 + 25 mg/day and doxazosin 4 mg.

A gastroscopy performed the day after the hospitalization revealed a congestive gastropathy with mild blood-vessels dilatation at the fundus, a small antral pseudopolipus, removed for histological exam and an ulcer at the upper duodenal knee. Some tumoral markers were mildly elevated: carcino-embrionary antigen (CEA) = 5.8 UI/L (0 - 4), CA 19.9 = 62 UI/L (0 - 37), tissue specific polipeptide (TPS) = 243 UI/L (0 - 80). Therapy with proton pump inhibitor was promptly started. The result of colonoscopy was not conclusive due to an inadequate colon cleaning. A second colonoscopy revealed only diffuse mucosal venous reticule accentuation and sigmoid diverticulosis. In exit, small, not congested hemorrhoids. A small bowel follow-through with capsule-endoscopy had normal findings.

Routine chest x-ray showed minimal pleural bilateral effusion with modeste disventilation at the lung basis. A total body CT scan evidenced centimetral lymphonodes at the mediastinum and at the aorto-pulmonary window, Barety lodge and prevascular. Subcentimetral lymphonodes between cava and aorta and in the mesenterial fan, high liver and spleen volumes were also found.

The fecal occult blood, searched in more occasions, was negative. Reticulocyte count and erythropoietin levels were in the normal ranges. After the normalization of hemoglobin levels, the patient was discharged with the recommendation of periodical hemato-biochemical controls.

A second gastroscopy was performed a month after the treatment with the proton pump inhibitor showing a small erosion at the gastric antrus. The hemoglobin levels were always low in spite of cyclical supplementation with intravenous iron, B_12_ vitamin and folate. Because of mild renal failure (Creatinin = 2.4 g/dL, Crea clearance = 27 ml/min, urinary protein loss = 10.2 gr/24h, glomerulary filtration rate GFR = 27 ml/min), and persistent anemia (Hb = 7.8 g/dL), he was treated with intramuscular beta-epoetin injections every 4 weeks. After three months, due to the continuous anemia despite these supplementations, new gastroscopy and colonoscopy were programmed. The gastroscopy had normal results; the colonoscopy finally, revealed diffuse, small angio-ectatic vessels in the colon, one of which with signs of recent bleeding.

After having identified the cause of bleeding, due to the high surgical risk and the multiple localizations, medical therapy was attempted.

The patient started subcutaneous octreotide 0.1 mg three times daily and switched to the long-acting release form of octreotide (LAR octreotide) 30 mg intramuscular every 28 days, two weeks later. The subcutaneous doses of 0.1 mg three times a day were continued for another two weeks. Moreover an oral dose of the beta-blocker atenolole 100 mg/day was added.

He continued LAR octreotide on a trial basis for six months. While on octreotide, the patient did not require transfusions nor iron, folate and B_12_ vitamin supplementation. Eritropoietin administration was gradually reduced too.

Hemoglobin levels stably reached levels higher than 12.0 mg/dL. The monthly hematological profile, including serum iron and ferritin and the fecal occult blood test were normal. Tumoral markers returned to the normal values.

The endoscopic evaluation at the end of 1-year follow-up showed disappearance of the smaller vascular lesions or significant reduction in size of those larger than 5 mm.

Moreover, no side effects, particularly those concerning cardiovascular events, abdominal pain, diarrhoea, changing in glycemic profile and development of cholelithiasis were recorded.

**Figure 1:**
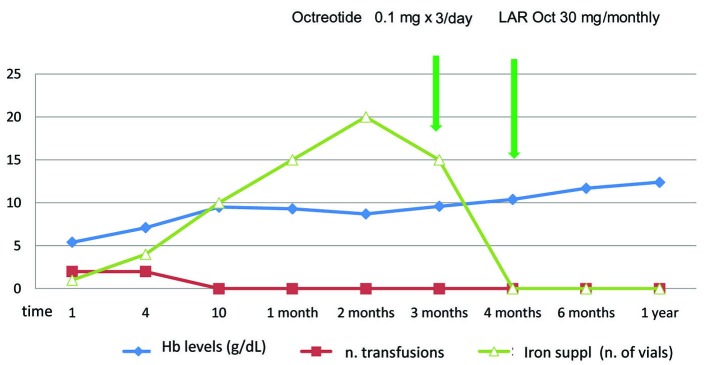
The trends of hemoglobin levels and needs of transfusion and parenteral iron supplementation before, during and after octreotide and LAR octreotide administration.

Figure 1 shows the trends of hemoglobin levels, needs of transfusion and parenteral iron supplementation before, during and after octreotide and LAR octreotide administration.

## Discussion

Degenerative AEs are commonly referred to as angiodysplasias and are a significant source of hemorrhage in patients over 60 years of age [2]. Boley et al suggested that degenerative AEs result from partial obstructions of submucosal veins while they traverse the longitudinal and circular layers of the muscolaris propria. The resulting pressure gradient may lead to incompetence of the precapillary sphincter and the development of arteriovenous communication [6].

The diagnostic evaluation of patients with obscure gastrointestinal bleeding includes endoscopy of the upper and lower gastrointestinal tract, radionuclide scintigraphy, and enteroscopy. As a general rule, the clinician should proceed from the simplest and least invasive tests to the more complex and invasive procedures [3]. If necessary, a repeat esophago-gastro-duodenoscopy and colonoscopy by an experienced endoscopist are mandatory. The colonoscopy should follow meticulous cleansing of the colon to allow detailed inspection of the mucosa for AEs. Excessive insufflations of the cecum and ascending colon could blanche angioectasias and should be carefully avoided because they are slow to refill [7]. AEs can also be difficult to detect in the presence of severe anemia and transfusion before endoscopy may augment the diagnostic yield [7].

In our case report other diagnostic hypothesis had to be excluded, for example, the presence of hepatosplenomegaly, multiple abdominal and thoracic lymphoadenopathies at the total body CT scan, might have raised suspicion of a lymphoproliferative disorder.

Moreover, anemia is common in patients with chronic heart failure (HF), being related to various factors including hemodilution, activation of the inflammatory cascade in chronic disease, urinary losses of serum erythropoietin and transferrin and associated renal insufficiency. HF itself stimulates the production of inflammatory cytokines such as interleukins 1, 6, 18 and tumor necrosis factor alpha (TNF-α), which have been reported to cause anemia in vitro [8]. Cytokine-mediated responses include reduced red blood cell progenitor proliferation with erythropoietin blunting, hepcidin elevation with resultant stunted iron intestinal absorption, and blockade of iron release from macrophages [9, 10]. ACE inhibitor use has also been implicated in the anemia related to HF by inhibiting erythroid precursor cells [11].

Our patient presented with combined cardiac and renal dysfunction, both negatively influencing anemia.

Among the possible pharmacological options to stop the bleeding from AEs are estrogen and progesteron, but, besides the lack of clear evidence of benefit, they can cause prohibitive side effects such as thrombosis [12]. The less systematically studied but second most common agent used is the somatostatin analogue, octreotide. The cyclic octapeptide octreotide is a somatostatin analogue producing the same effects on splanchnic hemodynamic with a longer biological half-life [13]. The theoretical effects of somatostatin/octreotide and why it may be useful in the therapy of bleeding AE vessels in different sites include: reduction of intestinal blood flow, improved platelet aggregation, inhibition of neoangiogenesis, possible increase of VWF levels and inhibition of gastric acid secretion [5, 14].

In a small uncontrolled study, it has been reported to prevent recurrent bleeding from gastrointestinal AEs when given subcutaneously at a dose of 0.1 mg three times a day for 6 months [14, 15] and in a more recent cohort study when administered at a lower dose for at least 1 year [5]. However, the need for multiple daily subcutaneous administrations represents a major drawback potentially limiting its extensive application. Monthly administration of long-acting formulation of octreotide (LAR-OCT) has been shown to provide similar effects compared to s.c. octreotide three times a day resolving two major concerns linked to the daily subcutaneous administration, fluctuating blood levels of the drug and the poor compliance of the patients [5]. Therefore, we believe that such a formulation should be preferred when a chronic treatment is the choice.

More serious effects of long-term octreotide may be related to glucose control in diabetic patients, and formation of gallstones or diarrhea due to the inhibition of pancreatic secretions [16].

A theoretical concern also includes a possible effect on myocardial function, and induction of pulmonary hypertension with heart failure [17]. Our patient did not present worsening of his preexisting heart and renal failure and glycemic control. At the same way, and according to the literature, no serious adverse events on renal and cardiac function have been observed.

Discontinuing octreotide should also be considered. Few data exist regarding chronic AE-related bleeding and the effect of pharmacological therapy. If treatment has been successful, some authors suggest to stop after six months of therapy and restart if bleeding recurs [5, 13]. According to Junquera et al, the probability of absence of rebleeding after 2 years of follow-up of patients receiving placebo was reported to be 36% [13].

In conclusion, our case report adds evidence that long-term monthly administration of LAR-OCT may be useful as a rescue therapy in controlling chronic bleeding from AE vessels in patients who cannot undergo surgery for reasons of old age, coexisting diseases and/or widespread localization of the lesions. The lack of adverse events, the better compliance because of monthly administration, and the comfortable home care with certain improvement in the quality of life represent additional advantages of LAR-OCT. Multicentric double-blind randomized trials are needed to support the efficacy of LAR-OCT therapy, identifying at the same time the appropriate dosage regimen and time of administration in different settings of patients.
